# Short tandem repeat expansions are a novel genetic risk factor for Alzheimer’s disease

**DOI:** 10.1002/alz.089952

**Published:** 2025-01-03

**Authors:** Michael H Guo, Wan‐Ping Lee, Badri N Vardarajan, Gerard D. Schellenberg, Jennifer E Phillips‐Cremins

**Affiliations:** ^1^ University of Pennsylvania Perelman School of Medicine, Philadelphia, PA USA; ^2^ Columbia University Medical Center, New York, NY USA

## Abstract

**Background:**

Studies of the genetics of Alzheimer’s disease (AD) have largely focused on single nucleotide variants and short insertions/deletions. However, much of the disease heritability has yet to be uncovered, suggesting that other forms of genetic variation promote substantial portions of genetic risk. Uncovering the genetic basis of AD can lead to new disease biomarkers and delineate disease mechanisms. As there are over one million short tandem repeats (STRs) in the genome and pathogenic expansions of STR cause over 30 neurologic diseases, it is important to ascertain whether STRs may also be implicated in AD risk.

**Method:**

Here, we utilize PCR‐free whole genome sequencing data from 2,981 individuals (1,489 AD case and 1,492 control individuals) to study the role of STRs in AD. We applied two cutting‐edge algorithms, ExpansionHunter and gangSTR, to genotype 321,742 STR tracts across the genome using the whole genome sequencing data. For each STR, we identified individuals carrying an expansion defined as having an STR tract length that is an outlier from the population. We then calculated the number of STR expansions in each individual and tested for differences in aggregate burden of STR expansions in AD case versus control individuals.

**Result:**

Individuals with AD had a 1.19‐fold increase burden of STR expansions compared to controls (p = 8.27 × 10^‐3^, two‐sided Mann Whitney test). Individuals carrying > 30 STR expansions had 3.62‐fold higher odds of having AD and had more advanced spread of tau neuropathology as measured by Braak staging. AD STR expansions were highly enriched within gene regulatory elements active in the hippocampus.

**Conclusion:**

These results demonstrate that harboring a high burden of STR expansions in the genome increases risk of AD and that STR expansions may serve as a novel genomic biomarker of AD risk and neuropathologic progression. Our findings suggest that STRs may disrupt gene expression and underscore the pivotal role of genomic instability in the onset and progression of AD.

